# Association of pericoronary fat attenuation index and insulin resistance for the risk of cardiometabolic multimorbidity: a cross-sectional study

**DOI:** 10.3389/fendo.2026.1801280

**Published:** 2026-04-21

**Authors:** Linjuan Li, Hui Li, Yuxin Guo, Yawen Wang, Ning Yang, Haimei Du, Zhuanxia Li

**Affiliations:** 1 Department of General Medicine, Yan’an University Affiliated Hospital, Yan’an, Shaanxi, China; 2 Department of Child Health Care, Northwest Women’s and Children’s Hospital, Xi’an, Shaanxi, China; 3 Department of Endocrinology, Yan’an University Affiliated Hospital, Yan’an, Shaanxi, China; 4 Department of Respiratory Medicine, Yan’an University Affiliated Hospital, Yan’an, Shaanxi, China

**Keywords:** cardiometabolic multimorbidity, cross-sectional study, pericoronary fat attenuation index, triglyceride-glucose index, type 2 diabetes mellitus

## Abstract

**Background:**

Cardiometabolic multimorbidity (CMM) has become an increasingly serious public health problem. Patients with type 2 diabetes mellitus (T2DM) often present with multiple cardiometabolic disorders and carry a significantly higher risk of CMM. Insulin resistance (IR) is the core mechanism of T2DM and atherosclerotic cardiovascular disease. The triglyceride-glucose index (TyG index) can serve as a reliable alternative for evaluating IR. The pericoronary fat attenuation index (FAI) is a non-invasive biomarker of coronary inflammation based on coronary CT angiography. However, the combined associations of the TyG index and FAI with CMM among patients with T2DM remain unknown. Therefore, this study aims to evaluate the TyG index, RCA-FAI, LAD-FAI, and LCX-FAI in relation to CMM among middle-aged and elderly patients with T2DM in China.

**Method:**

We conducted a cross-sectional study and enrolled 497 middle-aged and elderly patients (aged ≥45 years) with T2DM who underwent coronary CT angiography for clinical indications. We defined CMM as the concurrent presence of T2DM together with coronary heart disease or stroke. We used a multivariate logistic regression model to analyze the association between the TyG index and the FAI in each coronary segment (including RCA-FAI, LAD-FAI, and LCX-FAI) with CMM. We presented the study results as odds ratios (ORs) with their corresponding 95% confidence intervals (CIs). We employed restricted cubic splines to analyze the nonlinear relationship and used receiver operating characteristic (ROC) curves to assess the discriminatory capacity of each index in identifying CMM.

**Result:**

After fully adjusting for confounding factors, the TyG index (OR = 2.07, 95% CI: 1.44-2.99), RCA-FAI (for each increase of 1 unit: OR = 1.19, 95% CI: 1.14-1.23), LAD-FAI (OR = 1.16, 95% CI: 1.12-1.21), and LCX-FAI (OR = 1.11, 95% CI: 1.07-1.15) were all significantly and positively associated with CMM (all *P* < 0.001).Dosage-response analysis revealed nonlinear associations of the TyG index and LAD-FAI with CMM (*P* for nonlinearity < 0.05), whereas RCA-FAI and LCX-FAI showed linear relationships. Receiver operating characteristic (ROC) curve analysis was further performed to evaluate the discriminatory performance of each indicator for CMM. Among these indices, adding the RCA-FAI showed the most pronounced improvement, with a C-statistic of 0.900 (95% CI: 0.873–0.926, *P* < 0.001), a net reclassification improvement (NRI) of 0.749 (95% CI: 0.585–0.913, *P* < 0.001), and an integrated discrimination improvement (IDI) of 0.141 (95% CI: 0.110–0.171, *P* < 0.001). In contrast, adding the TyG index did not meaningfully improve the predictive value of the baseline clinical model.

**Conclusion:**

This study confirms that among middle-aged and elderly Chinese patients with T2DM, both the TyG index and FAI, including RCA-FAI, LAD-FAI, and LCX-FAI, are independently and positively associated with CMM. However, only coronary FAI indices significantly improve the discriminatory capacity for CMM, with RCA-FAI showing the strongest association and incremental predictive value. These findings suggest that FAI could serve as a useful imaging biomarker for identifying CMM status in patients with T2DM.

## Graphical Abstract

**Figure:**
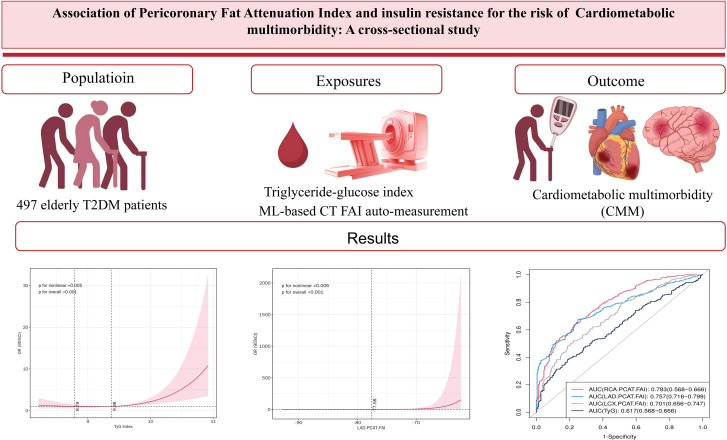
This cross-sectional study included 497 elderly patients with type 2 diabetes to explore the association between pericoronary fat attenuation index (FAI) and cardiometabolic multimorbidity (CMM). FAI was automatically quantified from coronary CT images using a machine learning-based technique. Nonlinear analyses demonstrated a significant positive association between FAI and the TyG index, a marker of insulin resistance. ROC curve analysis showed that FAI alone or combined with conventional indicators achieved moderate-to-good predictive performance for CMM. These results suggest that CT-derived FAI may serve as a promising imaging biomarker for evaluating cardiometabolic risk in elderly patients with type 2 diabetes.

## Introduction

Cardiometabolic multimorbidity (CMM) is defined as the coexistence of two or more cardiometabolic diseases, including coronary heart disease, diabetes mellitus, and stroke (1). Its global prevalence is rising rapidly, posing an increasingly severe public health burden (2). Studies have shown that CMM significantly increases the all-cause mortality risk of patients, shortens life expectancy, and seriously impairs quality of life (1, 3). This issue is particularly prominent among the middle-aged and elderly population in China. Epidemiological investigations show that the prevalence of CMM among people aged 45 and above can be as high as 24.5% (4). A large number of studies have further confirmed that CMM is closely related to multiple adverse outcomes, such as increased all-cause mortality, decreased cognitive function, and deteriorated quality of life among the elderly, constituting a significant public health problem in China (5–8).

Type 2 Diabetes Mellitus (T2DM) is one of the most common chronic diseases worldwide, and patients often have complications (9). Studies have shown that patients with T2DM as their first metabolic abnormality have a significantly increased cumulative risk of CMM and all-cause mortality (10). Therefore, accurately assessing and identifying T2DM individuals with CMM is clinically important.

Insulin resistance (IR) is the core pathophysiological mechanism underlying the development and progression of T2DM and atherosclerosis, and it is also an important risk factor for atherosclerotic cardiovascular diseases and stroke (11–13). Accumulating evidence indicates that the triglyceride-glucose (TyG) index is a reliable surrogate marker of insulin resistance (IR) and has been widely used for risk stratification and prognostic prediction in patients with T2DM and atherosclerotic cardiovascular disease (ASCVD) (14). Previous studies have confirmed that the TyG index is significantly associated with the risk of coronary heart disease (CHD) (15), stroke (16), and diabetes (17). Furthermore, high TyG index levels and their dynamic trajectories of increase and fluctuation are associated with an increased risk of CMM, and this association is particularly significant in the middle-aged population (18, 19).

The fat attenuation index (FAI) around the Coronary arteries is a measurement based on Coronary Computed Tomography Angiography (CCTA). Non-invasive biomarkers for evaluating the inflammatory level of Pericoronary Adipose Tissue (PCAT) (20, 21). Our previous research has confirmed that right coronary artery FAI is closely associated with the severity of coronary artery disease (22). In addition, increased attenuation of PCAT in the left anterior descending branch is not only independently associated with the risk of cardiovascular events but also provides additional risk stratification information for patients with T2DM (23). Further investigation reveals that FAI independently forecasts major adverse cardiovascular and cerebrovascular incidents in individuals with diabetes. This value exceeds that of traditional clinical features and other CCTA imaging findings (24, 25).

Although researchers have investigated insulin resistance indices in the context of CMM, to our knowledge, no study has simultaneously examined the combined associations of the triglyceride-glucose (TyG) index—a validated marker of systemic insulin resistance—and the fat attenuation index (FAI)—a reliable marker of local vascular inflammation—with CMM in any population. Therefore, this study aimed to investigate the associations of the TyG index and coronary artery FAI with the presence of CMM in a middle-aged and elderly Chinese population with T2DM. We hypothesized that higher TyG index and FAI levels would be independently associated with higher odds of CMM. Elucidating these associations may yield clinically accessible biomarkers to identify individuals with CMM in this population.

## Patients and methods

### Research design and population

**Figure 1:**
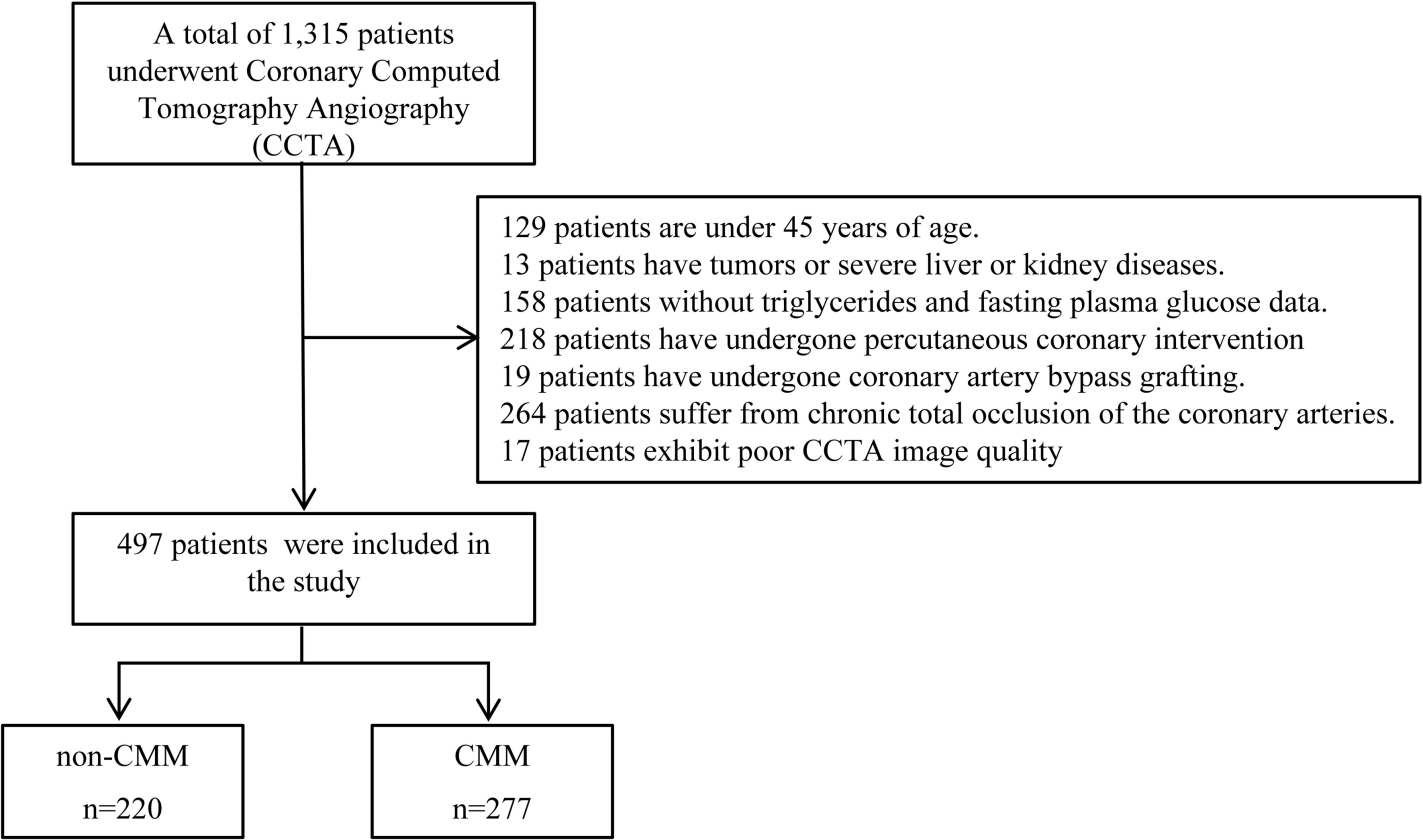
Flow chart of inclusion and exclusion criteria of participants. Flowchart depicting patient selection for a coronary computed tomography angiography (CCTA) study: 1,315 patients initially considered. Exclusion criteria listed for age, comorbidities, missing data, interventions, chronic occlusion, and poor image quality, leaving 497 included. These were divided into non-CMM (two hundred twenty) and CMM (two hundred seventy-seven) groups.

From September 2023 to September 2025, the Affiliated Hospital of Yan’an University enrolled 1315 middle-aged and elderly patients with T2DM for CCTA due to clinical reasons. We excluded the following types of patients (1): those under 45 years old; (2) Suffering from tumors or severe liver or kidney diseases; (3) Lack of triglycerides (TG) and fasting plasma glucose (FPG) data; (4) Have undergone percutaneous coronary intervention (PCI) or coronary artery bypass grafting (CABG);(5) Suffering from chronic total occlusion of coronary arteries (CTO); (6) Patients with poor CCTA image quality due to respiratory motion artifacts and irregular heart rate. We finally included 497 patients in this study. The flowchart of patient recruitment and study design is presented in **Figure 1**.

This study was performed in accordance with the Declaration of Helsinki and approved by the Ethics Committee of the Affiliated Hospital of Yan’an University. Written informed consent was obtained from all participants.

### Coronary CT angiography and analysis

All CCTA images produced by the “Siemens” company were acquired using a 256 CT scanner (SOMATOM Definition Flash, Siemens Healthcare, Munich, Germany). To ensure image quality, patients need to maintain calm breathing and keep their resting heart rate below 70 beats per minute. Patients with a resting heart rate above the target value should take 25 to 75 mg of a beta-blocker orally 1 hour prior to the examination. We inserted an 18–20 gauge intravenous cannula into the right median cubital vein and connected it to a dual−barrel power injector. We administered 60–80 mL of nonionic contrast medium iodixanol (320 mgI/mL) at an injection rate of 4.5–6.5 mL/s, adjusted according to patient body weight. For patients weighing more than 75 kg, we used iohexol (350 mgI/mL) instead. All scans were acquired in a retrospectively ECG−gated mode. The specific scanning parameters are as follows: The tube voltage is 120 kV, the tube current is 250 to 800 mA, the scanning interval is 0.18 mm(using the intelligent sector selection scanning mode), the rack rotation time is 270 ms, the collimator width is 128×0.625 mm, the matrix size is 512×512, the reconstruction interval is 0.5 mm, and the layer thickness is 0.75 mm (26).

In this study, we used the “Perivascular Fat Analysis Tool” software (Shukun Technology, Shukun (Beijing) Network Technology Co., Ltd., China) to examine the coronary arteries, including the left anterior descending artery (LAD) and the left circumflex artery (LCX). FAI was analyzed for the proximal 40−mm segment of the LCX and the proximal 10–50−mm segment of the RCA (26).

Two senior cardiovascular radiologists independently and blindly evaluated all CCTA results, and during the evaluation, the physicians were unaware of the patients’ clinical data, laboratory results, and CMM grouping status.

### Evaluation of the CMM incident

CMM is usually defined as an individual suffering from two or more cardiometabolic diseases simultaneously, such as diabetes, coronary heart disease, or stroke. The CMM status of all patients was determined based on the following objective criteria: 1)T2DM was defined as fasting blood glucose ≥7mmol/L, or 2-hour plasma glucose ≥11.17mmol/L as per the oral glucose tolerance test, or HbA1c≥6.5%, or current use of antidiabetic drugs, or self-reported history of diabetes. 2) CHD was defined based on CCTA findings. Two senior cardiovascular radiologists, blinded to clinical data, independently evaluated all images. CHD was considered present when at least one major epicardial artery (left main trunk, left anterior descending artery, left circumflex artery, or right coronary artery) exhibited luminal diameter stenosis ≥50%. It is important to emphasize that although both CHD diagnosis and FAI measurement were derived from the same CCTA examination, they reflect distinct pathophysiological processes: CHD diagnosis is based on structural assessment of luminal narrowing, whereas FAI serves as a functional biomarker quantifying inflammation in pericoronary adipose tissue, thereby providing information potentially independent of the stenosis severity. 3) Stroke is defined as an ischemic stroke event confirmed through an electronic inpatient medical record system, characterized by acute neurological deficits and confirmed by brain CT or MRI imaging. Within the framework of this study, those who met the conditions of T2DM and simultaneously met either CHD or stroke were classified into the CMM group. Otherwise, they will be classified into the non-CMM group (i.e., only the T2DM group). Two researchers independently extracted disease information from the electronic inpatient medical record system, and inconsistencies were checked.

### TyG calculation and definition of key indicators

In this study, the TyG index was calculated using the following formula: ln[fasting triglycerides (mg/dL) × fasting blood glucose (mg/dL)/2]. The units of triglycerides (TG) and fasting plasma glucose (FPG) in the original test data are mmol/L, which have been converted to international units (FPG: 1 mmol/L = 18 mg/dL; TG: 1 mmol/L = 88.57 mg/dL). The formula for calculating Body Mass Index (BMI) is weight (kg) divided by height (m) squared. Hypertension (HTN) is defined as: According to the “2024 Chinese Hypertension Prevention and Treatment Guidelines”, it is diagnosed with a systolic blood pressure of ≥140 mmHg and a diastolic blood pressure of ≥90 mmHg, or as having been diagnosed with hypertension and receiving treatment. ② Definition: Definition of smoking: A lifetime cumulative or continuous smoking history, smoking at least 20 cigarettes per day for at least 6 months.

### Statistical analysis

We categorized participants according to CMM status and used the Kolmogorov–Smirnov test to assess the normality of continuous variables. Normally distributed variables were presented as mean and standard deviation, whereas skewed variables were presented as median and interquartile range. Categorical variables are expressed as frequencies (percentages). Test for differences among different CMM status groups: Chi-square test was used for categorical variables; For continuous variables, the independent sample t-test (for normal distribution) or the Wilcoxon rank sum test (for two independent samples that are not normally distributed) is selected based on their distribution characteristics. Multivariable logistic regression models were used to evaluate the associations of the TyG index, RCA-FAI, LAD-FAI, and LCX-FAI with CMM, respectively. The results were expressed as odds ratios (ORs) and their 95% confidence intervals (CIs). The hierarchical regression model is constructed as follows: Model 1 is unadjusted; Model 2 adjusts for age and gender; Model 3 further adjusts for smoking status, body mass index (BMI), hypertension, glycated hemoglobin A1c (HbA1c), total cholesterol (TC), low-density lipoprotein cholesterol (LDL), high-density lipoprotein cholesterol (HDL), and the use of antidiabetic agents, lipid-lowering agents, and aspirin. To further investigate the dose−response relationship among TyG index, FAI, and CMM, restricted cubic spline (RCS) functions were applied to perform nonlinear association analyses with adjustment for covariates in Model 3. Four nodes were set, and the Akaike information criterion (AIC) was used to optimize the model fitting. Receiver operating characteristic (ROC) curves were plotted, and the area under the curve (AUC) was calculated to assess the discriminatory ability of the indicators for identifying CMM. Differences in AUC between models were compared using the DeLong test. Furthermore, the Net Reclassification Index (NRI) and Integrated Discrimination Improvement (IDI) were calculated to quantify the incremental predictive value of the TyG index, RCA-FAI, LAD-FAI, and LCX-FAI beyond established risk factors. Based on age (65 years or ≥65 years), gender (male or female), body mass index (BMI, 24 kg/m², ≥24 kg/m² and < 28 kg/m² or ≥28 kg/m²), hypertension status (yes or no), and blood glucose control level (glycated hemoglobin ≥7% or <; Stratified subgroup analysis was conducted for 7%) to further evaluate the potential interaction effect between FAI and CMM. All statistical analyses in this study were performed using IBM SPSS Statistics version 26.0 and the Free Statistical Analysis Platform (Version 2.1.1, Beijing, China; http://www.clinicalscientists.cn/freestatistics). All statistical tests were two-sided, and *P* values < 0.05 were considered statistically significant.

## Result

### Study the population and baseline characteristics

A total of 497 patients with coronary artery disease were included in this study. The median age was 63.0 years (57.0-71.0 years), among which 62.17% were male. The average TyG index of the study population was 9.42 ± 0.64. The average RCA-FAI was -80.89 ± 8.18 HU, and the medians of LAD-FAI and LCX-FAI were -77.62 HU and -69.95 HU, respectively.

Compared with the non-CMM group, patients in the CMM group were older, had a higher proportion of males, a higher proportion of smokers, a higher HbA1c level, and a higher use rate of lipid-lowering drugs and aspirin (all *P* < 0.05). However, the levels of BMI, HDL, and LDL in the CMM group were lower (P < 0.05). There were no significant differences between the two groups in systolic blood pressure, diastolic blood pressure, fasting blood glucose, total cholesterol, triglycerides, apolipoprotein A, and apolipoprotein B levels (all *P* >0.05). Among the leading research indicators, the TyG index, RCA-FAI, LAD-FAI, and LCX-FAI in the CMM group were significantly higher than those in the non-CMM group (all P < 0.001; **Table 1**).

**Table 1:** Demographic and clinical characteristics.

Characteristics	Total	Non-CMM	CMM	Statistic	*P value*
	N= 497	N= 220	N= 277		
Age,year	63.00 (57.00, 71.00)	61.50 (56.00, 70.00)	65.00 (59.00, 71.00)	Z=-3.26	0.001
Gender, n(%)				χ²=4.57	0.032
Male	309 (62.17)	129 (57.08)	180 (66.42)		
Female	188 (37.83)	97 (42.92)	91 (33.58)		
Smoking, n(%)	242 (48.69)	81 (35.84)	161 (59.41)	χ²=27.40	<0.001
BMI,kg/m2	24.87 (23.05, 26.73)	25.31 (23.29, 26.77)	24.54 (22.94, 26.46)	Z=-2.23	0.026
SBP,mmHg	136.00 (128.00, 147.00)	135.00 (125.50, 149.00)	136.00 (128.00, 146.00)	Z=-0.35	0.729
DBP,mmHg	80.00 (74.00, 86.00)	81.00 (75.00, 86.75)	80.00 (74.00, 85.00)	Z=-1.47	0.142
FbG,mmol/L	7.30 (6.30, 8.98)	7.09 (6.30, 8.54)	7.41 (6.34, 9.32)	Z=-1.70	0.089
HbA1c ,%	7.40 (6.70, 8.50)	7.15 (6.60, 8.10)	7.60 (6.80, 8.97)	Z=-3.75	<0.001
TG ,mmol/L	4.07 (3.40, 5.01)	4.27 (3.48, 5.17)	3.98 (3.34, 5.00)	Z=-1.69	0.092
TC ,mmol/L	1.75 (1.28, 2.48)	1.70 (1.24, 2.37)	1.76 (1.29, 2.49)	Z=-0.57	0.569
HDL,mmol/L	1.01 (0.87, 1.16)	1.04 (0.90, 1.18)	0.99 (0.86, 1.15)	Z=-2.55	0.011
LDL,mmol/L	1.98 (1.50, 2.66)	2.09 (1.57, 2.81)	1.90 (1.42, 2.53)	Z=-2.33	0.020
ApoA,g/L	1.25 (1.08, 1.44)	1.26 (1.09, 1.42)	1.25 (1.08, 1.44)	Z=-0.09	0.930
ApoB,g/L	0.67 (0.52, 0.82)	0.70 (0.54, 0.83)	0.65 (0.51, 0.82)	Z=-1.57	0.117
TyG Index	9.42 ± 0.64	9.27 ± 0.54	9.54 ± 0.68	t=-4.93	<0.001
FAI,HU					
RCA	-80.89 ± 8.18	-85.38 ± 7.06	-77.32 ± 7.20	t=-12.49	<0.001
LAD	-77.62 (-82.50, -72.28)	-80.66 (-85.00, -77.19)	-74.23 (-79.05, -69.88)	Z=-9.86	<0.001
LCX	-69.95 (-75.00, -65.00)	-72.50 (-78.09, -68.00)	-67.48 (-72.24, -63.29)	Z=-7.71	<0.001
Basal chronic disease, n (%)					
Hypertension	381 (77.13)	168 (75.00)	213 (78.89)	χ²=1.05	0.306
Medications,n(%)					
Antidiabetic	404 (81.29)	187 (82.74)	217 (80.07)	χ²=0.58	0.447
Antidyslipidemic	427 (85.92)	161 (71.24)	266 (98.15)	χ²=73.78	<0.001
Aspirins	323 (64.99)	109 (48.23)	214 (78.97)	χ²=51.17	<0.001

Data are presented as n (%), median (interquartile range, IQR), or mean ± standard deviation (SD). CMM, Cardiometabolic Multimorbidity; BMI, body mass index; SBP, Systolic Blood Pressure; DBP, Diastolic Blood Pressure; FBG, Fasting Blood Glucose; HbA1c, Glycosylated Hemoglobin A1c; TC, Total Cholesterol; LDL, Low-Density Lipoprotein; TG, Triglycerides; HDL, High-Density Lipoprotein;ApoA, Apolipoprotein A; ApoB, Apolipoprotein B; TyG, triglyceride-glucose index; PCAT, pericoronary adipose tissue; FAI, fat attenuation index; RCA, right coronary artery; LAD, left anterior descending coronary artery; LCX, left circumflex coronary artery. P values were derived from Chi-square test, Mann-Whitney U test (Z), or independent t-test as appropriate.

### Population characteristics stratified based on the TyG index

To deeply explore the relationship between insulin resistance and patient characteristics, we divided the population into four quantile arrays (Q1-Q4) based on the TyG index, with the cutoff values being: Q1< 8.99, Q2: 8.99-9.38, Q3: 9.38-9.81, Q4 ≥ 9.81. Compared with group Q1, the levels of systolic blood pressure, fasting blood glucose, HbA1c, triglycerides, total cholesterol, LDL, apolipoprotein B, as well as the values of RCA-FAI, LD-FAI, LCX-FAI, and the prevalence of CHD and CMM in groups Q2-Q4 were all higher. In contrast, HDL levels were lower (all P < 0.05). However, there were no significant differences among the TyG quantile arrays for gender, smoking status, diastolic blood pressure, apolipoprotein A level, hypertension prevalence, and rates of hypoglycemic drug and aspirin use (all P > 0.05; **Table 2**).

**Table 2:** Baseline characteristics of participants stratified by TyG index quartiles.

Characteristics	TyG index				Statistic	P
	Q1 (<8.99)	Q2 (≥8.99,<9.38)	Q3 (≥9.38,<9.81)	Q4 (≥9.81)		
Participants,NO	124	124	124	125		
Age,year	64.50 (59.00,71.00)	66.50 (60.00,72.00)	62.00 (56.00,70.00)	61.00 (56.00,69.00)	χ²=8.44	0.038
Gender, n(%)					χ²=4.88	0.181
Male	76 (61.29)	84 (67.74)	68 (54.84)	81 (64.80)		
Female	48 (38.71)	40 (32.26)	56 (45.16)	44 (35.20)		
Smoking, n(%)	54 (43.55)	68 (54.84)	53 (42.74)	67 (53.60)	χ²=6.15	0.104
BMI,kg/m2	24.79 (22.98,26.61)	24.42 (23.03,26.12)	25.39 (23.87,27.34)	24.80 (23.03,26.79)	χ²=6.37	0.095
SBP,mmHg	134.00 (122.00,140.00)	136.00 (129.00,146.50)	137.00 (126.00,146.00)	138.00 (130.00,150.00)	χ²=11.05	0.011
DBP,mmHg	80.00 (72.00,85.00)	80.00 (75.00,86.00)	81.00 (74.00,86.00)	81.00 (75.00,87.00)	χ²=4.24	0.236
FBG,mmol/L	6.14 (5.60,6.70)	7.07 (6.50,8.00)	7.50 (6.70,8.70)	10.30 (8.40,12.42)	χ²=223.42	<0.001
HbA1c ,%	6.70 (6.20,7.20)	7.20 (6.60,8.03)	7.40 (6.70,8.33)	8.80 (7.90,9.90)	χ²=140.43	<0.001
TG ,mmol/L	3.77 ± 0.86	4.44 ± 1.00	4.50 ± 1.03	4.74 ± 1.09	F=21.72	<0.001
TC ,mmol/L	1.21 ± 0.29	1.69 ± 0.29	2.42 ± 0.51	3.65 ± 1.26	F=275.35	<0.001
HDL,mmol/L	1.06 ± 0.23	1.07 ± 0.27	1.01 ± 0.24	0.99 ± 0.21	F=3.14	0.025
LDL,mmol/L	1.90 ± 0.77	2.38 ± 0.77	2.38 ± 0.80	2.55 ± 0.79	F=16.00	<0.001
ApoA,g/L	1.26 (1.06,1.43)	1.26 (1.07,1.44)	1.25 (1.13,1.43)	1.24 (1.10,1.46)	χ²=0.57	0.902
ApoB,g/L	0.56 (0.48,0.67)	0.66 (0.54,0.82)	0.72 (0.56,0.81)	0.79 (0.63,0.92)	χ²=47.68	<0.001
FAI,HU						
RCA	-87.15 ± 7.18	-82.89 ± 6.19	-79.76 ± 6.77	-73.81 ± 6.16	F=90.44	<0.001
LAD	-79.95 ± 7.96	-77.63 ± 6.67	-76.97 ± 6.76	-75.54 ± 7.38	F=8.11	<0.001
LCX	-73.20 ± 8.27	-70.39 ± 7.76	-69.73 ± 7.02	-67.76 ± 5.77	F=11.94	<0.001
Basal chronic disease, n (%)						
CHD, n(%)	47 (37.90)	55 (44.35)	53 (42.74)	78 (62.40)	χ²=17.27	<0.001
Stroke, n(%)	16 (12.90)	21 (16.94)	26 (20.97)	31 (24.80)	χ²=6.40	0.094
CMM, n(%)	55 (44.35)	66 (53.23)	65 (52.42)	91 (72.80)	χ²=22.13	<0.001
Hypertension	93 (75.00)	91 (73.39)	98 (79.67)	99 (79.20)	χ²=2.01	0.570
Medications,n(%)					χ²=0.15	0.985
Antidiabetic	104 (83.87)	92 (74.19)	103 (83.06)	105 (84.00)	χ²=5.51	0.138
Antidyslipidemic	92 (74.19)	102 (82.26)	112 (90.32)	121 (96.80)	χ²=29.68	<0.001
Aspirins	76 (61.29)	80 (64.52)	80 (64.52)	87 (69.60)	χ²=1.94	0.585

Data are presented as n (%), median (interquartile range, IQR), or mean ± standard deviation (SD). BMI, body mass index; SBP, Systolic Blood Pressure; DBP, Diastolic Blood Pressure; FBG, Fasting Blood Glucose; HbA1c, Glycosylated Hemoglobin A1c; TC, Total Cholesterol; LDL, Low-Density Lipoprotein; TG, Triglycerides; HDL, High-Density Lipoprotein; ApoA, Apolipoprotein A; ApoB, Apolipoprotein B; TyG, triglyceride-glucose index; PCAT, pericoronary adipose tissue; FAI, fat attenuation index; RCA, right coronary artery; LAD, left anterior descending coronary artery; LCX, left circumflex coronary artery; CHD,Coronary Heart Disease; CMM, Cardiometabolic Multimorbidity.P values for continuous variables were derived from one-way ANOVA (normally distributed) or the Kruskal-Wallis test (non-normally distributed, reported as Chi-square statistic). P values for categorical variables were derived from the Chi-square test.

### The association of the TyG index with coronary artery inflammation and CMM

To evaluate the independent associations of the TyG index, RCA-FAI, LAD-FAI, and LCX-FAI with CMM, multivariable logistic regression models were constructed (**Table 3**). The model adopts a step-by-step adjustment strategy: Model 1 remains unadjusted; Model 2 corrects demographic factors (age, gender); Model 3 further corrects for smoking status, body mass index (BMI), hypertension, glycated hemoglobin A1c (HbA1c), total cholesterol (TC), low-density lipoprotein cholesterol (LDL), high-density lipoprotein cholesterol (HDL), and the use of antidiabetic agents, lipid-lowering agents, and aspirin.

**Table 3:** LogisticLogistic regression analyses for the associations of PCAT parameters and TyG index with CMM.

Characteristics	Model 1		Model 2		Model 3	
	OR (95%CI)	*P* value	OR (95%CI)	*P value*	OR (95%CI)	*P* value
RCA PCAT FAI (per SD increase)	1.17 (1.13 ~ 1.21)	<0.001	1.18 (1.14 ~ 1.22)	<0.001	1.19 (1.14 ~ 1.24)	<0.001
Quartiles of RCA PCAT FAI						
Quartile 1	1.00 (Reference)		1.00 (Reference)		1.00 (Reference)	
Quartile 2	1.40 (0.84 ~ 2.33)	0.192	1.49 (0.88 ~ 2.50)	0.135	1.38 (0.76 ~ 2.35)	0.354
Quartile 3	2.32 (1.40 ~ 3.85)	0.001	2.29 (1.36 ~ 3.87)	0.002	2.96 (1.71 ~ 4.12)	<0.001
Quartile 4	3.45 (2.03 ~ 5.85)	<0.001	3.37 (1.95 ~ 5.81)	<0.001	3.34 (1.73 ~ 6.10)	<0.001
LAD PCAT FAI (per SD increase)	1.16 (1.12 ~ 1.20)	<0.001	1.16 (1.12 ~ 1.20)	<0.001	1.16 (1.12 ~ 1.21)	<0.001
Quartiles of LAD PCAT FAI						
Quartile 1	1.00 (Reference)		1.00 (Reference)		1.00 (Reference)	
Quartile 2	1.03 (0.62 ~ 1.72)	0.897	1.04 (0.68 ~ 1.85)	0.965	1.03 (0.63 ~ 1.80)	0.473
Quartile 3	1.80 (1.09 ~ 2.97)	0.023	1.75 (1.05 ~ 2.92)	0.033	1.72(1.03 ~ 3.74)	0.039
Quartile 4	3.40 (2.03 ~ 5.76)	<0.001	3.36 (1.91 ~5.83)	<0.001	3.07 (1.81 ~ 5.23)	<0.001
LCX PCAT FAI (per SD increase)	1.12 (1.08 ~ 1.15)	<0.001	1.12 (1.08 ~ 1.15)	<0.001	1.10 (1.06 ~ 1.14)	<0.001
Quartiles of LCX PCAT FAI						
Quartile 1	1.00 (Reference)		1.00 (Reference)		1.00 (Reference)	
Quartile 2	1.49 (0.90 ~ 2.48)	0.123	1.52 (0.90 ~ 2.57)	0.118	1.48 (0.86 ~ 3.05)	0.113
Quartile 3	1.79 (1.08 ~ 2.95)	0.023	1.77 (1.06 ~ 2.97)	0.03	1.60 (0.85 ~ 3.01)	0.143
Quartile 4	3.38 (2.00 ~ 5.71)	<0.001	3.40 (1.96 ~ 5.88)	<0.001	2.65 (1.75 ~ 3.60)	<0.001
TyG Index (per SD increase)	2.03 (1.50 ~ 2.75)	<0.001	2.30 (1.67 ~ 3.16)	<0.001	1.92 (1.21 ~ 3.04)	0.006
Quartiles of TyG						
Quartile 1	1.00 (Reference)		1.00 (Reference)		1.00 (Reference)	
Quartile 2	1.43 (0.87 ~ 2.35)	0.163	1.40 (0.84 ~ 2.34)	0.197	1.43 (0.87 ~ 2.35)	0.163
Quartile 3	1.38 (0.84 ~ 2.28)	0.204	1.64 (0.98 ~ 2.75)	0.062	1.38 (0.84 ~ 2.28)	0.204
Quartile 4	3.36 (1.98 ~ 5.70)	<0.001	3.90 (2.25 ~ 6.75)	<0.001	3.36 (1.98 ~ 5.70)	<0.001

CMM, Cardiometabolic Multimorbidity; TyG, triglyceride-glucose index; OR,Odds Ratio; CI, Confidence IntervalPCAT, pericoronary adipose tissue; FAI, fat attenuation index; RCA, right coronary artery; LAD, left anterior descending artery; LCX, left circumflex artery.

Model 1: Unadjusted.

Model 2: Adjusted for age and gender.

Model 3: Adjusted for age, gender, smoking status, body mass index (BMI), hypertension, glycated hemoglobin A1c (HbA1c), total cholesterol (TC), low-density lipoprotein cholesterol (LDL), high-density lipoprotein cholesterol (HDL), and the use of antidiabetic agents, lipid-lowering agents, and aspirin.

In fully corrected Model 3, the TyG index and all FAIs were significantly positively correlated with CMM: the TyG index (OR = 1.92, 95%CI:1.21-30.4, *P* = 0.006), RCA-FAI (increase per unit: OR = 1.19, 95% CI: 1.14-1.24, P < 0.001), LAD-FAI (increase per unit: OR = 1.16, 95% CI: 1.12-1.21, *P* <; 0.001), LCX-FAI (increase per unit: OR = 1.10, 95% CI: 1.06-1.14, *P* < 0.001).

According to quantile analyses of each indicator, the likelihood of CMM increased with elevated quantiles of the TyG index and FAI. Using Q1 as the reference group, the highest risk of CMM was observed in the TyG index Q4 group (OR = 3.36, 95%CI: 1.98–5.70). For FAI, the risk of CMM was significantly increased in the RCA-FAI Q3 and Q4 groups (Q3: OR = 2.96, 95%CI: 1.71–4.12; Q4: OR = 3.34, 95%CI: 1.73–6.10). The highest risk of CMM was found in the Q4 groups of LAD-FAI and LCX-FAI (LAD-FAI Q4: OR = 3.07, 95%CI: 1.81–5.03; LCX-FAI Q4: OR = 2.65, 95%CI: 1.75–6.60).

**Figure 2:**
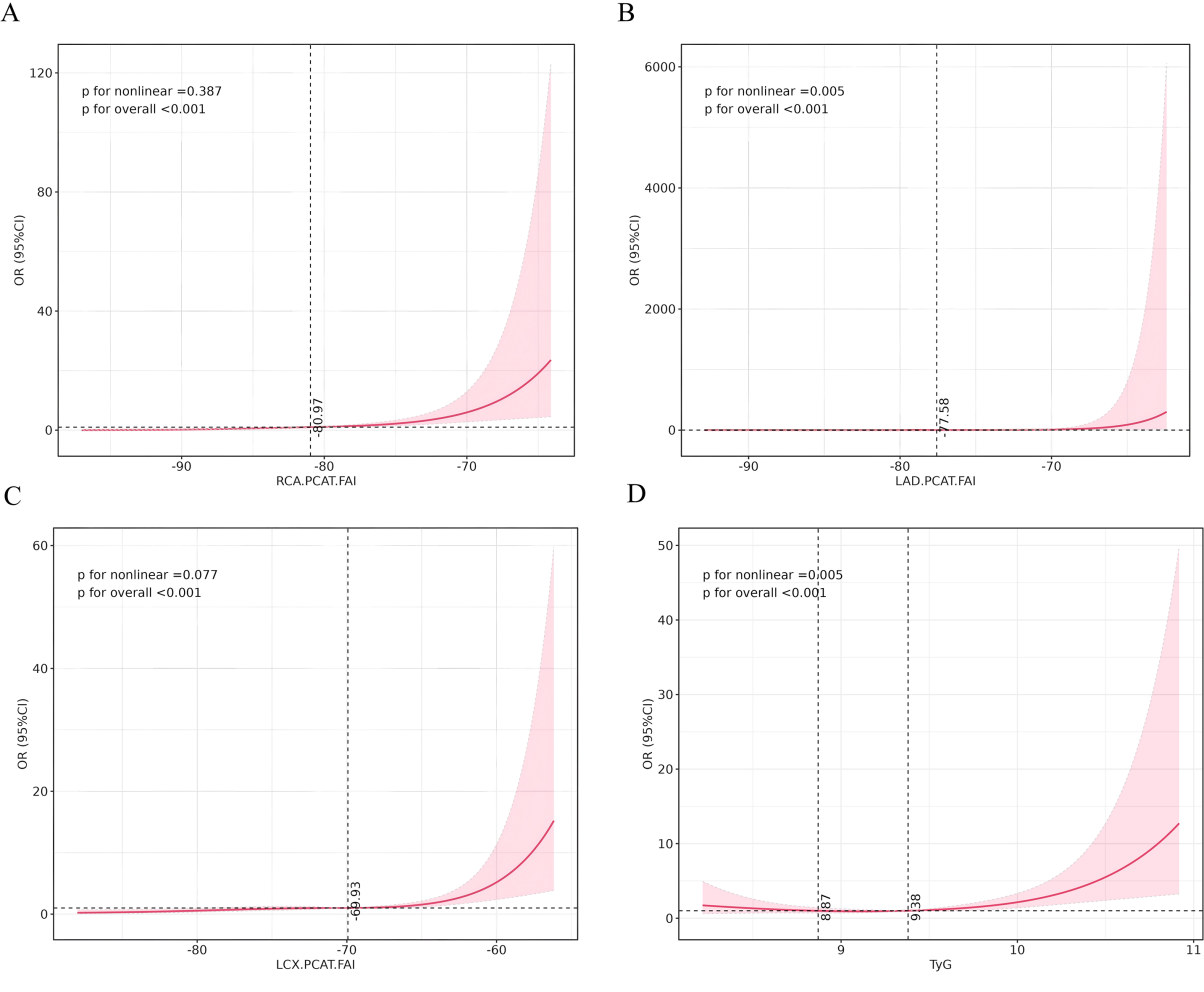
Analysis of the correlation between FAI, TyG index, and CMM using the restricted cubic spline (RCS) model. Restricted cubic spline curves for CMM were plotted for **(A)** RCA-PCAT FAI, **(B)** LAD-PCAT FAI, **(C)** LCX-PCAT FAI, and **(D)** TyG index after adjustment for confounders. The solid line shows the adjusted odds ratio, with the shaded area representing the 95% confidence interval. The model was adjusted for age, gender, smoking status, body mass index (BMI), hypertension, glycated hemoglobin A1c (HbA1c), total cholesterol (TC), low-density lipoprotein cholesterol (LDL), high-density lipoprotein cholesterol (HDL), and the use of antidiabetic agents, lipid-lowering agents, and aspirin.

To further explore the potential nonlinear relationship between the above indicators and CMM, we performed restricted cubic spline (RCS) analysis. After adjustment for covariates, the TyG index and LAD-FAI showed significant nonlinear associations with CMM (overall *P* < 0.001; *P* for nonlinearity=0.005), whereas RCA-FAI and LCX-FAI exhibited linear associations with CMM (overall *P* < 0.001; *P* for nonlinearity>0.05) (**Figure 2**).

### The incremental predictive value of the TyG index and coronary artery inflammation for CMM

**Figure 3:**
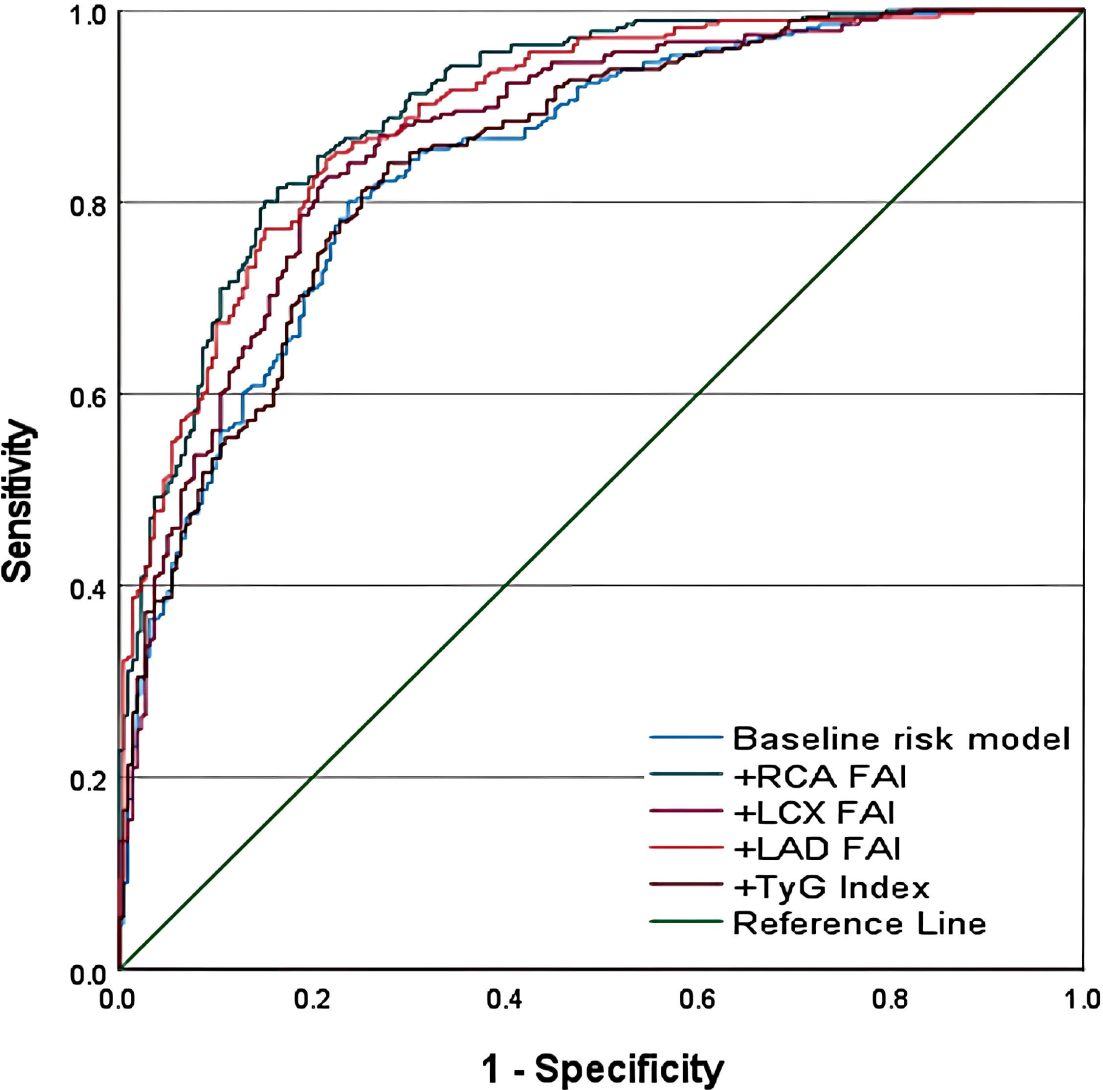
Flowchart depicting patient selection for a coronary computed tomography angiography (CCTA) study: 1,315 patients initially considered. Exclusion criteria listed for age, comorbidities, missing data, interventions, chronic occlusion, and poor image quality, leaving 497 included. These were divided into non-CMM (two hundred twenty) and CMM (two hundred seventy-seven) groups.

We constructed receiver operating characteristic (ROC) curves to evaluate the discriminatory performance of the baseline clinical model and the baseline clinical model combined with RCA-FAI, LAD-FAI, LCX-FAI, and the TyG index for CMM (**Figure 3**). Significant differences were observed between the baseline clinical model [0.842 (0.808–0.877), P<0.001] and the model incorporating RCA-FAI [0.900 (0.873–0.926)], LAD-FAI [0.890 (0.862–0.918)], or LCX-FAI [0.867 (0.835–0.899)] (all P<0.005). In contrast, no significant difference was found compared with the model including the TyG index [0.846 (0.812–0.879), P = 0.464].

**Table 4** summarizes the C-statistics, net reclassification improvement (NRI), and integrated discrimination improvement (IDI). For CMM prediction, the addition of RCA-FAI significantly improved model performance: NRI 0.749 (95% CI: 0.585–0.913), IDI 0.141 (95% CI: 0.110–0.171; all P<0.001). Similarly, LAD-FAI and LCX-FAI also significantly enhanced predictive performance (all P<0.001 for NRI and IDI). In comparison, the TyG index did not meaningfully improve predictive ability: the slight increases in NRI and IDI (both P<0.05) were clinically negligible.

**Table 4:** Incremental predictive value of coronary inflammation indicators for CMM.

Model	C-statistic (95% Cl)	*P* value	NRI (95% CI)	*P* value	IDI (95% CI)	*P* value
Baseline risk model	0.842(0.808-0.877)	Ref	Ref		Ref	
+RCA-FAI	0.900(0.873-0.926)	<0.001	0.749(0.585-0.913)	<0.001	0.141(0.110-0.171)	<0.001
+LAD-FAI	0.890(0.862-0.918)	<0.001	0.690(0.523-0.857)	<0.001	0.112(0.085-0.140)	<0.001
+LCX-FAI	0.867(0.835-0.899)	0.004	0.388(0.205-0.553)	<0.001	0.057(0.037-0.077)	<0.001
+TyG Index	0.846(0.812-0.879)	0.464	0.312(0.137-0.487)	0.004	0.011(0.001-0.021)	0.027

NRI, net reclassification improvement; IDI, integrated discrimination improvement; CI, confidence interval; The Baseline risk model included age, gender, smoking status, body mass index (BMI), hypertension, glycated hemoglobin A1c (HbA1c), total cholesterol (TC), low-density lipoprotein cholesterol (LDL), high-density lipoprotein cholesterol (HDL), and the use of antidiabetic agents, lipid-lowering agents, and aspirin.

The baseline clinical model included age, sex, smoking status, body mass index (BMI), hypertension, HbA1c, TC, LDL, HDL, and the use of antidiabetic agents, lipid-lowering drugs, and aspirin.

### Subgroup analysis

Stratified analysis was conducted by age, gender, BMI, smoking status, hypertension, and blood glucose control (**Table 5**). The results showed that only in the subgroup of blood glucose control was there a significant interaction between the TyG index and FAI and CMM (*P* for interaction < 0.05); no significant interaction was observed in the other subgroups (all *P* for interaction > 0.05).

**Table 5:** Subgroup Analysis of the Association between PCAT FAI and TyG Index with CMM.

	No		RCA PCAT FAI		LAD PCAT FAI		LCX PCAT FAI		TyG Index	
Characteristics	Non-CMM	CMM	OR (95%CI)	*P* for interaction	OR (95%CI)	*P* for interaction	OR (95%CI)	*P* for interaction	OR (95%CI)	*P* for interaction
All patients	220	277	1.17 (1.13 ~ 1.21)		1.15 (1.11 ~ 1.19)		1.11 (1.08 ~ 1.15)		1.71 (1.23 ~ 2.37)	
Age,year				0.946		0.686		0.922		0.559
<60	90	75	1.18 (1.11 ~ 1.26)		1.14 (1.08 ~ 1.21)		1.12 (1.06 ~ 1.19)		2.14 (1.20 ~ 3.83)	
≥60	130	202	1.17 (1.12 ~ 1.23)		1.16 (1.11 ~ 1.21)		1.11 (1.07 ~ 1.16)		1.76 (1.15 ~ 2.68)	
Sex				0.682		0.178		0.395		0.187
Male	126	183	1.16 (1.11 ~ 1.21)		1.13 (1.09 ~ 1.18)		1.10 (1.06 ~ 1.14)		1.49 (1.00 ~ 2.20)	
Female	94	94	1.18 (1.12 ~ 1.26)		1.21 (1.13 ~ 1.29)		1.14 (1.08 ~ 1.20)		2.34 (1.25 ~ 4.35)	
BMI,kg/m^2^				0.284		0.690		0.060		0.405
<24	65	116	1.23 (1.14 ~ 1.33)		1.17 (1.10 ~ 1.25)		1.18 (1.10 ~ 1.26)		1.38 (0.75 ~ 2.55)	
≥24	155	161	1.15 (1.10 ~ 1.20)		1.15 (1.10 ~ 1.20)		1.09 (1.05 ~ 1.13)		1.97 (1.32 ~ 2.94)	
Smoking				0.960		0.131		0.461		0.904
No	143	112	1.17 (1.12 ~ 1.23)		1.21 (1.14 ~ 1.28)		1.13 (1.07 ~ 1.18)		1.66 (1.05 ~ 2.63)	
Yes	77	165	1.17 (1.11 ~ 1.23)		1.13 (1.08 ~ 1.18)		1.10 (1.05 ~ 1.15)		1.81 (1.10 ~ 2.97)	
HBP				0.934		0.628		0.521		0.332
No	56	59	1.18 (1.10 ~ 1.26)		1.14 (1.06 ~ 1.22)		1.15 (1.07 ~ 1.23)		1.19 (0.54 ~ 2.61)	
Yes	163	218	1.17 (1.13 ~ 1.22)		1.16 (1.11 ~ 1.21)		1.11 (1.07 ~ 1.15)		1.85 (1.29 ~ 2.67)	
HbA1c,%				0.562		0.205		0.287		0.004
<7	92	85	1.20 (1.12 ~ 1.28)		1.20 (1.12 ~ 1.29)		1.15 (1.08 ~ 1.22)		0.62 (0.30 ~ 1.28)	
≥7	128	191	1.16 (1.11 ~ 1.21)		1.14 (1.09 ~ 1.19)		1.10 (1.06 ~ 1.14)		2.25 (1.47 ~ 3.46)	

CMM, Cardiometabolic Multimorbidity; OR, odds ratio; CI, confidence interval; PCAT, pericoronary adipose tissue; FAI, fat attenuation index; RCA/LAD/LCX, right coronary artery/left anterior descending artery/left circumflex artery; TyG, triglyceride-glucose index; BMI, body mass index; HBP, hypertension; HbA1c, glycated hemoglobin.

All models within subgroups are adjusted for medication use where not used as the stratification variable. The P value for interaction tests whether the strength of association differs significantly between subgroups.

## Discussion

This study demonstrates that, after comprehensive adjustment for confounders, both the TyG index and coronary inflammation indicators (RCA-FAI, LAD-FAI, and LCX-FAI) are independently associated with CMM. Specifically, nonlinear associations were identified for the TyG index and LAD-FAI, while linear relationships were observed for RCA-FAI and LCX-FAI. Moreover, incremental predictive analyses demonstrated that incorporating coronary inflammation—most notably RCA−FAI—significantly enhanced the discriminatory performance of the baseline model for CMM, whereas adding the TyG index provided no substantial incremental benefit.

PCAT, a component of pericardial adipose tissue directly surrounding the coronary arteries, functions as an active endocrine and paracrine organ. The PCAT attenuation index, a functional imaging marker of coronary inflammation, has been extensively investigated, and the right coronary artery FAI (RCA-FAI) has been identified as a key biomarker for early detection of this inflammation (20). A lesion-specific pericoronary FAI higher than -83.5 HU (HR = 2.017,95% CI 1.143-3.559, p = 0.015) was an independent prognostic factor for MACE in patients with T2DM (24). A higher LAD-PCAT index can predict cardiovascular events in patients with T2DM (23). Accumulating evidence confirms that FAI not only predicts cardiovascular events but is also closely associated with the coexistence of CMM. Consistent with these findings, RCA-FAI, LAD-FAI, and LCX-FAI were all independently and positively associated with CMM in the present study. Notably, incremental predictive analysis further highlighted that RCA-FAI exhibited superior discriminatory value for CMM compared with the TyG index and LCX-FAI, suggesting that the PCAT attenuation index is a valuable, easily measurable biomarker for identifying high-risk individuals.

The RCA is considered the optimal anatomical site for evaluating coronary inflammation (27, 28). Its location within the right atrioventricular groove offers anatomical stability and minimal interference with cardiac motion, conferring superior reproducibility to RCA-FAI measurements (29). Accumulating evidence indicates that both RCA-FAI and PCAT volume are independent predictors of CHD (30). These findings are in line with our previous research demonstrating that coronary inflammation, assessed by RCA-FAI, partially mediates the proatherogenic effect of the atherogenic index of plasma (AIP) on multivessel coronary artery disease (MVCAD) (22). Collectively, These findings confirm that RCA-FAI is a reliable biomarker, independently associated with CHD, independent of glycometabolic status, and further support its superior incremental predictive value for CMM observed in the present study.

The TyG index is a reliable alternative indicator for evaluating insulin resistance (IR), which is the common core pathogenesis of T2DM, metabolic syndrome, and atherosclerotic cardiovascular disease (ASCVD). Studies have found that a higher TyG index is significantly associated with an increased risk of T2DM. For every 1-unit increase in the TyG index, the risk of T2DM significantly increases (14, 31). Furthermore, the study found that a higher TyG index was significantly associated with the risk of CHD (the fully adjusted CHD hazard ratio was 2.32 (95% CI 1.16-4.68, p-trend 0.04)), and the TyG index was an independent risk factor for CHD (32). The TyG index is an independent predictor of all-cause mortality and MACE in patients with CMM (coexisting with CHD and T2DM) (33). The HR associated with high TyG index and stroke prevalence analysis was 1.25 (95% CI: 1.06-1.47) (34). Consistent with the above findings, this study found that the TyG index is positively correlated with CMM.

RCS analysis showed a significant nonlinear association between the TyG index, LAD-FAI, and CMM risk. In contrast, RCA-FAI and LCX-FAI showed a linear relationship with CMM. Dose-response meta-analysis showed that the association between TyG index and stroke was nonlinear (35). A study found a “U-shaped” association between the TyG index and mortality in patients with CMM (36). This study also made similar findings. There was a U-shaped association between the TyG index and the likelihood of CMM, with the strength of this association being lowest when the TyG index was between 8.79 and 9.38. Future research is necessary to explore further the mechanism of this nonlinear dose-response relationship among insulin resistance, coronary artery inflammation, and CMM.

The results of this study support the theoretical framework of “systemic metabolic disorder - local vascular inflammation - aggregation of cardiac metabolic diseases”. The potential mechanisms may involve multiple approaches. A high TyG index indicates that the body is in a state of hyperinsulinemia, hyperglycemia, and dyslipidemia. These factors act jointly on the cardiovascular system, inducing endothelial dysfunction, an oxidative stress response, and a pro-inflammatory cascade, thereby promoting the development and progression of atherosclerosis and its complications (37–39). Chronic low-grade inflammation is a key pathophysiological driver connecting IR and CMM. In the context of insulin resistance and systemic inflammation, adipose tissue around the coronary arteries undergoes phenotypic transformation. The secretion of pro-inflammatory factors (such as tumor necrosis factor-α, interleukin-6, and MCP-1) increases, while adiponectin secretion, which has protective effects, decreases (40, 41). The adipose tissue surrounding the inflamed coronary arteries directly aggravates damage to adjacent coronary arteries via paracrine signals. It regulates atherosclerosis by bidirectionally releasing inflammation-related mediators. The NF-κB and JAK/STAT pathways are core regulatory pathways that accelerate the progression of atherosclerotic plaques and increase plaque vulnerability. This pathological process can be reflected in an increase in the FAI value (42, 43). Therefore, FAI offers a window into vascular health beyond the mere anatomical information provided by luminal stenosis, which underlies our CHD definition. By demonstrating a positive association between FAI and CMM (which includes CHD), our findings suggest that the inflammatory milieu, as captured by FAI, is closely linked to the presence of structural disease, but the two are not circularly defined. Studies have shown that the TyG index, RCA-FAI, LAD-FAI, and LCX-FAI are all positively associated with CMM, indicating that insulin resistance and coronary artery inflammation markers are closely related to CMM and can serve as simple, effective indicators for evaluating CMM in this population. However, further institutional research is still needed to clarify the specific roles of these indicators in CMM development.

This research has several advantages. First, it simultaneously examined the TyG index, a surrogate marker of insulin resistance, and FAI, a non-invasive imaging biomarker of coronary inflammation, in relation to CMM in a Chinese middle-aged and elderly population with T2DM. Secondly, the research relies on CCTA, a commonly used clinical imaging technique. The FAI values used in this study are objective, quantifiable, and reproducible, providing a potentially clinically translatable imaging tool for identifying CMM. In addition, in statistical analysis, we not only analyzed the TyG index and FAI as continuous and categorical variables, but also used the RCS model to explore nonlinear associations, and conducted extensive subgroup analyses and interaction tests. These methods enhanced the robustness and reliability of the research results. However, this study also has several limitations. Firstly, the cross-sectional design of the study limits our ability to establish causal relationships among the TyG index, FAI, and CMM. The reported associations are correlational and require confirmation in prospective cohort studies. Secondly, this research was conducted as a single-center study. Importantly, all participants underwent CCTA due to clinical suspicion of coronary artery disease, which introduces selection bias. Our study cohort, therefore, represents a subgroup of T2DM patients with a higher pre-test probability of cardiovascular disease, rather than the general T2DM population. This is reflected in the relatively high prevalence of CMM in our sample (55.7%). Consequently, the findings may not be generalizable to community-dwelling individuals with T2DM without overt cardiovascular symptoms. The observed associations need validation in larger, more diverse, and population-based cohorts. Thirdly, although the Definition of CMM is based on objective clinical and Imaging criteria, the determination of stroke in it relies on inpatient medical records, and there may be cases of missed diagnosis of mild or unhospitalized cases. Finally, although we adjusted for multiple potential confounding factors, including demographic characteristics, traditional risk factors, and medication history in the multivariable model, there may still be unmeasured or not fully adjusted confounding variables (such as dietary habits, physical activity levels, other inflammatory markers, etc.) that affect our results.

## Conclusion

This study confirms that among middle-aged and elderly Chinese patients with T2DM, both the TyG index and FAI, namely RCA-FAI, LAD-FAI, and LCX-FAI, are independently and positively associated with CMM. However, predictive modeling revealed striking differences in their clinical utility: although the TyG index showed a nonlinear relationship with CMM, it did not significantly improve model performance. In contrast, coronary artery FAI indices significantly improved discrimination and reclassification ability, among which RCA-FAI showed the strongest association and incremental predictive value. Collectively, these findings suggest that coronary inflammation imaging offers superior clinical translational potential for CMM identification compared with conventional metabolic markers. Notably, RCA-FAI holds great promise as a key imaging biomarker for identifying T2DM patients at high risk of CMM.

## Data Availability

The study utilized existing clinical and imaging datasets from hospital records rather than prospectively collected data. Due to institutional privacy policies and patient confidentiality agreements, raw data cannot be shared upon request. Requests to access these datasets should be directed to ZL at lizhuanxia_000@163.com.
